# Three-Year Follow-Up of Participants from a Self-Weighing Randomized Controlled Trial

**DOI:** 10.1155/2017/4956326

**Published:** 2017-09-19

**Authors:** Lua Wilkinson, Carly R. Pacanowski, David Levitsky

**Affiliations:** ^1^Division of Nutritional Sciences, University of Alabama, Birmingham, Birmingham, AL, USA; ^2^Department of Behavioral Health and Nutrition, University of Delaware, Newark, DE, USA; ^3^Division of Nutritional Sciences, Cornell University, Ithaca, NY, USA

## Abstract

Frequent self-weighing is associated with weight loss maintenance. Several years ago, we investigated frequent self-weighing's effect on weight loss and found the participants lost a significant amount of weight. Three years after this trial's end, participants were contacted for an update on their weight and self-weighing frequency. Weight change and self-weighing frequency since the end of the study were assessed. We hypothesized that participants who maintained frequent self-weighing behavior would have maintained their weight loss. Out of 98 participants enrolled in the RCT, 37% (*n* = 36) participated in this follow-up study. Total weight loss during the trial for the follow-up participants was 12.7 ± 19.4 lbs (*p* < 0.001). Three years after intervention, participants regained 0.9 ± 4.34 lbs, a value that was not statistically different from zero (*p* = 0.75). This did not differ by gender (*p* = 0.655). Over 75% of these participants continued to weigh themselves at least once a week. Frequent self-weighing may be an effective, low-cost strategy for weight loss maintenance. Future research should further investigate the role of self-weighing in long-term weight gain prevention.

## 1. Introduction

The global increase in weight gain presents an urgent need for interventions that reverse this trajectory. Thousands of diet and exercise programs have been introduced that successfully reduce body weight of adults, yet none have translated to large-scale public health improvements [1–3]. The peak of weight loss from most behavioral modification interventions occurs at 3–6 months; after this, most individuals begin to regain some of the lost weight, even if the intervention continues [4–6].

Approximately one-quarter of adults in the United States are actively trying to lose weight, most of whom are doing so without professional support [7]. The Obesity Society, along with the American Heart Association and the American College of Cardiology, recommends overweight and obese adults lose weight through a calorie restriction of 500–750 kcal per day and the help of an intensive, comprehensive lifestyle program [8]. Long-term, sustained weight loss from these programs is a concern; less than one out of six adults who has ever been overweight or obese has maintained weight loss of at least 10% [9]. For weight loss efforts to be clinically and/or personally meaningful, emphasis should also be placed on effective long-term weight management strategies [10].

Frequent self-weighing may be effective for long-term weight loss maintenance [11–13]. However, data from self-weighing trials are frequently limited to the intervention period [14]. This paper presents weight outcomes 3 years after the end of an effective randomized controlled trial (active treatment versus no treatment) with delayed control focusing on slow and steady weight loss through frequent self-weighing and visual feedback, a process called the Caloric Titration Method [15].

## 2. Subjects and Methods

### 2.1. Intervention

**Figure 1:**
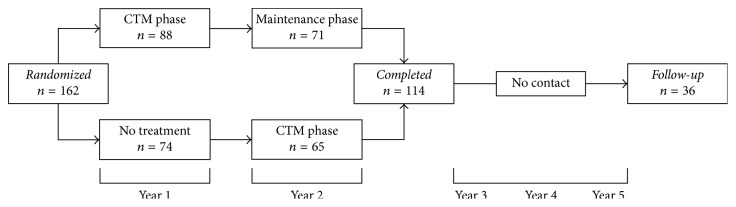
Overview of study design.

This study is a follow-up analysis of a two-year randomized controlled trial with delayed control, the Caloric Titration Method (CTM) weight loss trial [16], which ran between the fall of 2010 and the fall of 2012 (Figure 1()). One hundred sixty-two overweight or obese adults were randomly assigned to a 12-month daily self-weighing intervention (*n* = 88) or delayed control group (*n* = 74). The delayed control group received the intervention one year after the intervention group, at which time the intervention group was directed to maintain the weight they had lost. By the end of the 2-year trial, all participants received the same lifestyle modification program of daily self-weighing with automated, personalized electronic feedback, consisting of a graph of their weight over time. All participants were weighed in person at baseline and 6, 12, and 24 months to validate weight trends.

Inclusion criteria were adults over the age of 18 interested in losing weight and with body mass index (BMI) > 27.0 kg/m^2^. Excluded were those who were pregnant or planning on becoming pregnant, individuals who had diabetes, or those with a history of an eating disorder.

### 2.2. Intervention Outcomes

**Figure 2:**
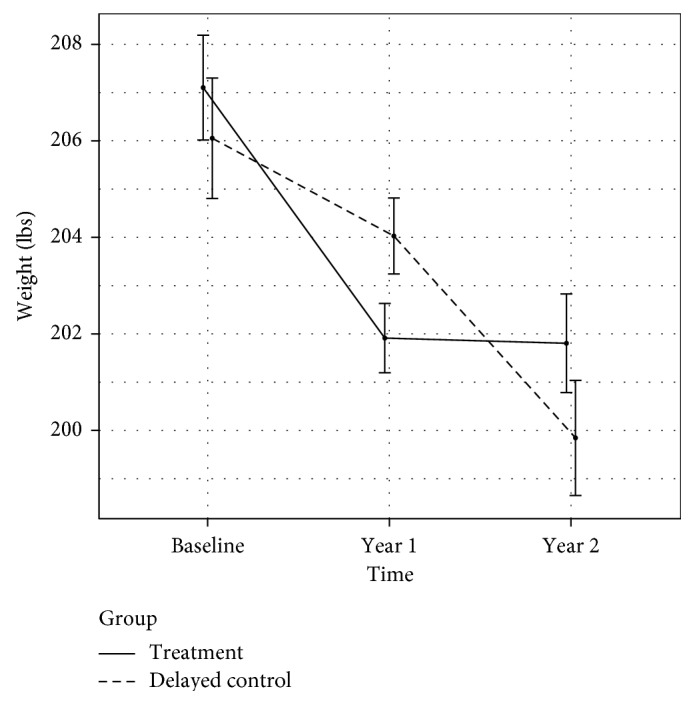
Weight change during the CTM trial.

At the end of the first year, the intervention group logged an average of 5.5 weights per week into the CTM website. The mean weight loss of this group was 5.72 ± 13.0 lbs, a significant difference from the mean weight loss of the delayed control, which was 1.1 ± 9.7 lbs (*p* = 0.019). During year 2, the delayed control group entered an average of 4.7 weights per week into the CTM system. The mean weight loss of the delayed control was 4.2 ± 12.5 lbs, a value not different from the mean weight loss of the intervention group in year 1 (*p* = 0.524). The intervention group's weight change during the 2nd year was 0.2 ± 10.6 lbs, a value not different from zero (*p* = 0.929), and they continued to weigh themselves an average of 4.5 times per week. After the 2-year intervention was completed, the total mean weight change was −6.6 ± 15.9 lbs for all participants [16] (Figure 2()). Frequency of weighing's effect on weight change was not reported in the original study; post hoc analysis showed no effect in either unadjusted (*p* = 0.68) or adjusted (adjusting for group randomization, gender, and baseline weight) models (*p* = 0.90).

### 2.3. Follow-Up Measures

Three years after the trial ended, in the summer of 2015, participants in both treatment and delayed control groups were contacted and asked if they were interested in providing updated weight measurements and filling out an online questionnaire similar to the one given in the study.

Questionnaires were emailed to those who consented to follow-up contact and included questions on self-weighing behaviors and the long-term utility of the CTM. Participants were asked to reenter their current body weight in the same way they had during the intervention.

## 3. Statistical Analysis

Characteristics for those who accepted the survey invitation are described by randomized group, gender, age, education level, ethnicity, baseline body weight and BMI, weight change, frequency of weighing during the study, and current self-reported body weight. Demographic and outcome data of those who agreed to the follow-up survey were compared to those who declined/did not respond using Student's *t* and *χ*^2^ tests. Weight loss maintenance (in lbs and % weight) was assessed using paired *t*-tests between the end of the trial and follow-up. Differences in gender were assessed using *χ*^2^ tests. Data cleaning and restructuring was done using SPSS version 23. Statistical analyses were performed in R 3.2.5 in the summer of 2016. *p* values < 0.05 were considered significant.

## 4. Results

### 4.1. Participant Characteristics

We obtained follow-up data from 36 participants (37% of those contacted), referred to hereafter as “respondents.” Of the original sample of 162, 56 (34%) did not consent to contact beyond the end of the study. One hundred six participants were emailed to assess interest in participating in a follow-up survey. Eight emails were returned as invalid. Of the 98 individuals that received the email, 40 responded: 36 were willing to provide follow-up information, and 4 were not.

Respondents had a mean age (±SD) of 53.09 ± 9.78 years and most self-identified as white (94%) (Table 1). Twenty-four respondents were from the treatment group (67%), and ten (28%) were male. The mean weight was 197.59 ± 39.31 lbs.

The mean weight loss during the two-year trial was 12.7 ± 19.4 lbs, and respondents weighed themselves an average of 6.14 ± 1.5 times per week during the “CTM phase” of the intervention (year 1 for those in the intervention group; year 2 in the delayed control group). Most individuals continued to weigh themselves at least once a week after the end of the CTM study (*n* = 27; 75%). Most respondents reported that they found participating in the CTM program helpful in reaching their goal weight in the long term (*n* = 35; 97%).

Compared to the larger sample of 98 individuals, there were no systematic differences between those who accepted the survey invitation and those who declined in terms of group assignment, baseline weight/BMI, gender, or age (Table 2). Nonetheless, those who agreed to the survey lost more weight during the study than those who did not respond or did not agree to the survey (12.7 ± 19.4 lbs versus 4.28 ± 14.17 lbs, *p* = 0.018).

There were also differences in frequency per week of weighing; survey responders weighed themselves more often during the CTM phase compared to those who did not respond (6.15 ± 0.76 times/week versus 4.87 ± 1.86 times/week, *p* ≤ 0.001).

The majority (97% in the nonresponders compared to 100% in the responders) weighed themselves weekly or more during the CTM phase; no differences were found when comparing survey responders and nonresponders in whether they weighed themselves at least weekly or more (*p* = 0.704).

### 4.2. CTM's Association with Long-Term Weight Loss Maintenance

**Figure 3:**
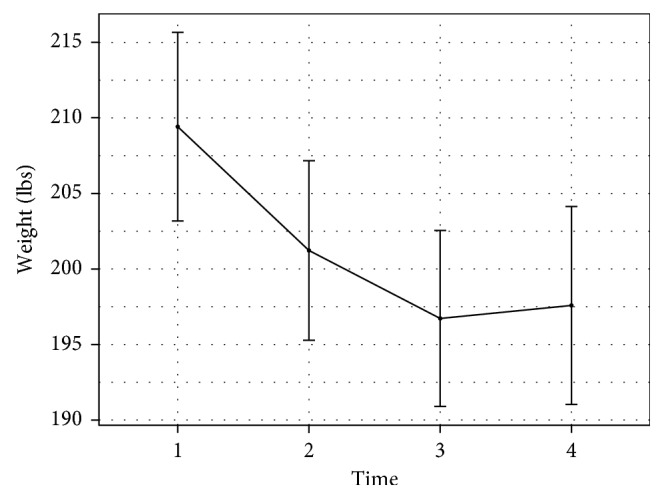
Weight change between baseline and follow-up.

Collectively, weight increased by 0.9 ± 16.4 lbs (0.46 ± 7.9%) since the end of the trial 3 years priorly. Paired *t*-tests show no significant difference between weight at the end of year 2 and weight at follow-up (*p* = 0.75). Weight change was not moderated by gender (*p* = 0.655). Thirteen individuals gained weight (>3% increase in weight), 16 maintained weight (±3% change, based on the published definition of weight maintenance [17]), and 7 lost weight (>3% decrease in weight); in total, 64% of respondents maintained or lost weight after the trial's end (see Figures 3() and 4() for weight change over time).

**Figure 5:**
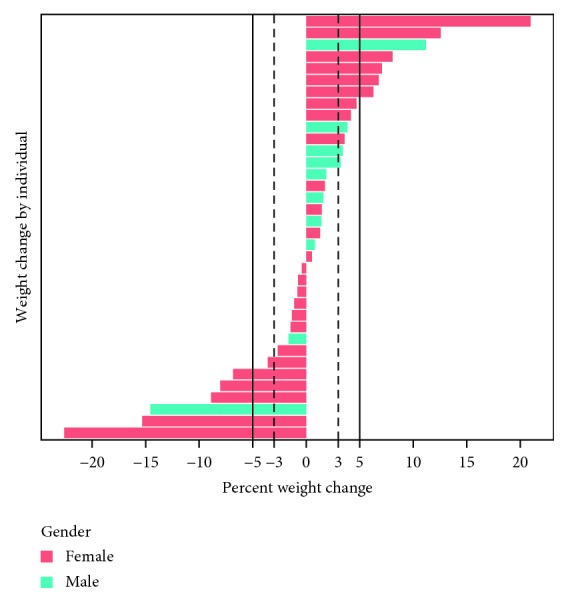
Individual weight changes.

Of those who lost at least 5% of their body weight at the end of the 2-year study (*n* = 17; 47% of respondents), 59% (*n* = 10; or 28% of all respondents) maintained their weight from the end of the trial (see Figure 5() for weight change by individual).

**Figure 6:**
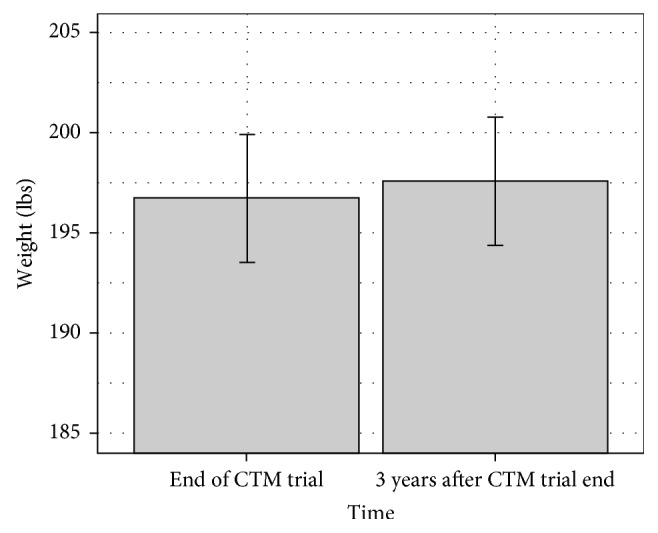
Weight change between end and follow-up.

The mean total weight change was −11.8 ± 24.9 lbs, or −5.3 ± 10.76%, from starting body weight five years priorly (Figure 6()).

**Figure 7:**
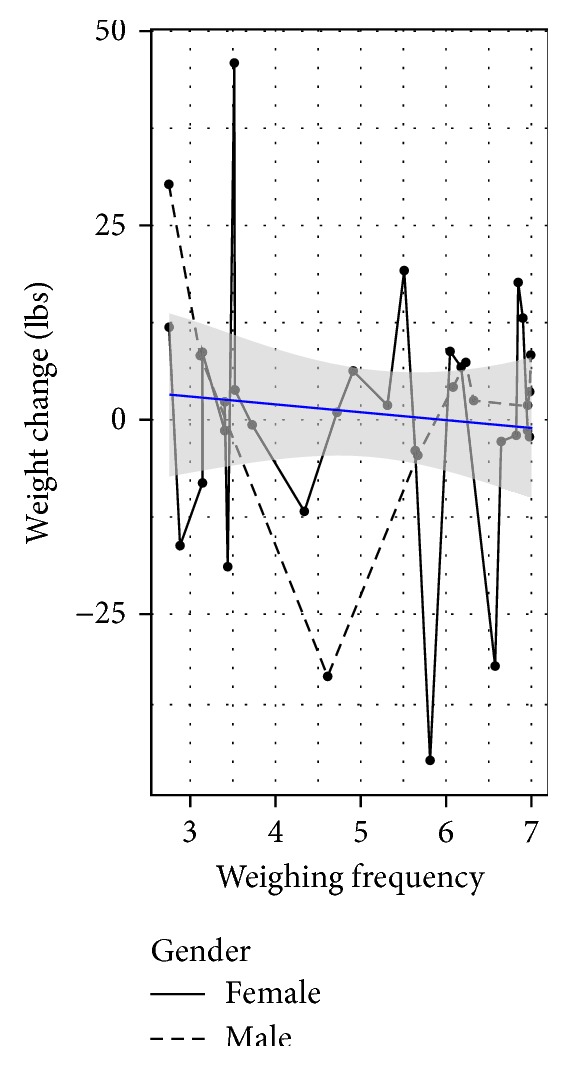
Weight change from the end of the study to follow-up as a function of how often the participant weighs themselves.

We looked for evidence of a linear trend between frequency of weighing during the CTM phase and weight 3 years later and found no association in an unadjusted regression model (*p* = 0.28) (Figure 7()). This finding was not qualitatively affected by adjusting for sex, baseline weight, or group assignment (*p* = 0.92). We also found no association between the current reported frequency of weighing (more or less than weekly) and weight maintenance (*p* = 0.325). This finding was not affected by adjusting for sex, baseline weight, or group assignment (*p* = 0.244).

## 5. Discussion

This analysis of 3-year weight loss maintenance indicates, on average, that the respondents who used the CTM maintained weight losses of 12.7 lbs, representing a reduction of ∼5% from starting body weight.

### 5.1. Long-Term Weight Loss Maintenance

Current evidence on long-term weight loss maintenance following an intervention has not been definitive. The current ACC/AHA/TOS guidelines for weight management report there is evidence that 40–60% of people participating in high-intensity, long-term, comprehensive weight loss interventions maintain at least a 5% weight loss after 2 years of follow-up after randomization [8]. However, our findings report outcomes 5 years after randomization of a low-intensity, noncomprehensive program. In our cohort, 10 individuals (28% of respondents) maintained weight losses of 5% or more; for individuals whose current weight is less than or equal to their weight at the end of the trial, this number increases to 44% of respondents (*n* = 16). Analysis of higher intensity programs that follow individuals longer than 2 years has shown similar results. In a meta-analysis of US adults who completed strict diet-induced weight loss programs, Anderson et al. [18] found that 32% of respondents maintained their weight losses 3 years after completing the weight loss program, although the median number of respondents available at follow-up (82%) was greater than for the current study (37%).

Closer examination of the responders' weight during the study shows that 9 (25%) of these individuals gained weight during the 2-year trial. Of these 9, seven maintained their weight 3 years later, while 2 continued to gain. Heterogeneity of individuals in weight loss trials has been noted by Kaiser and Gadbury [19], who estimated that as many as 30–40% of a sample may have a treatment effect in the opposite direction than the mean. While the overall mean of the responders in this trial was not different from zero, it is important to note that the variability of weight change is sizable, both during the trial and after.

The primary goal of the CTM trial was to promote weight loss. Yet, given the data presented here, as well as the fact that only 2 individuals out of 36 gained weight throughout the trial and follow-up period, self-weighing may play a role in weight gain prevention. Preventing weight gain, even for those who may already be overweight or obese, can have major public health implications. American adults gain an average of one pound per year between ages 20 and 65 [20, 21]; according to the most updated review on weight gain prevention interventions by the Agency for Healthcare Research and Quality, the majority of weight control trials continue to neglect weight gain prevention in favor of weight loss [22]. Of the trials that do explicitly address preventing weight gain, most do not follow participants after the cessation of the intervention, and study quality is poor. While only 28% of our respondents lost 5% or more during the two-year trial and kept it off for 3 years, 89% (*n* = 32) kept their weight within 3% of starting weight or less over a five-year period. Self-weighing's role in weight gain prevention is worth further exploration.

### 5.2. Frequency of Weighing and Weight Maintenance

Although frequent self-weighing has been shown to be a common strategy used by people successful in weight loss maintenance [23], the present data are among the few to examine long-term outcomes from self-weighing by itself, not part of a more comprehensive program. One reason this program may be useful for people to sustain the weight loss may lie within the nature of the CTM. The CTM electronically sets small weight loss goals (1% weight losses) and allows the participant to find the technique that works for them to reach those goals. Even though considerable day-to-day variations occur in their weight, over several days a pattern of weight change is exhibited in the graph illustrating to the participant the success of their energetic changes they made. The general idea of the CTM is not to provide individuals with specific instructions or diet on how to lose weight, but to allow individuals the autonomy to choose which method(s) works for them. They are free to change their diet one week and increase physical activity the next, for example. The CTM is a tool that encourages self-regulation through proximal feedback of weight outcomes, which gives an individual interested in weight control more precise information about how their behaviors are influencing their weight over the long term. In the same way, a control group might maintain or lose weight through being accountable to a research therapist [24], so may the person using the CTM become accountable to themselves.

Another property of the CTM that might account for its success in sustaining weight loss lies in its simplicity. Frequent weighing easily becomes a part of the morning awakening ritual. The participants were instructed to put the scale next to their bed and weigh themselves immediately after rising (after voiding). This procedure reduces variability of the weight due to clothing and, after a few weeks, becomes part of the early morning routine.

**Figure 8:**
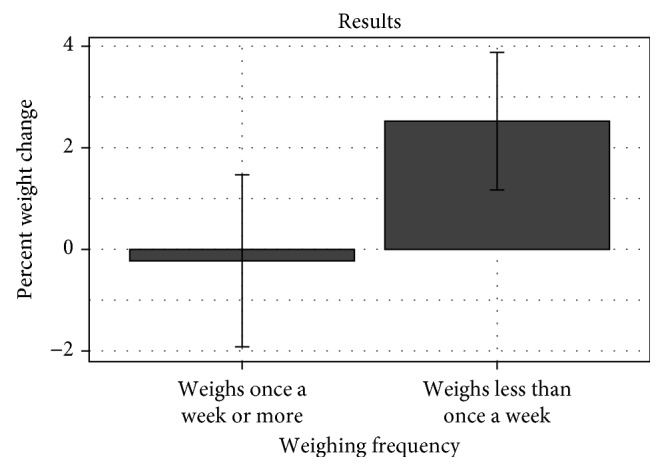
Percentage weight change as a function of weighing oneself weekly or more compared to weekly or less.

We are not able to identify what specific aspect of the CTM trial had the largest effect on weight. Since the study was designed to promote slow and steady weight loss through frequent weighing, we assessed weight maintenance and its association to self-weighing after intervention using survey responses. 22% of participants who responded to the follow-up reported that they continued to weigh themselves daily. While most reported that they weigh themselves once a week or more, the primary aim of the original intervention was to see if frequent weighing with feedback affected weight. Those who continued to weigh themselves once a week or more maintained a weight slightly below their weight at the end of the study (Figure 8()). This warrants further investigation, as it is possible that weekly weighing is as effective as other intervals for weight loss maintenance—other studies on frequent weighing have shown improvements in weight loss with weekly weighing [25–28].

This study has a number of limitations. First, comparisons with a control group cannot be made; respondents all received the CTM daily weighing intervention at the time of follow-up. However, a greater percentage of respondents maintained their weight loss when compared to participants in other intensive behavioral treatment studies [18]. Second, self-selection bias may be present in that survey respondents lost more weight during the study period than those who did not participate. Respondents also weighed themselves more frequently. This study had a lower response rate than other longitudinal follow-up studies [18]. This lower response rate, coupled with significant weight loss differences between those who responded, may influence the direction of the long-term weight loss results. Lastly, in order to maximize follow-up responses, we did not ask the participants to come in to be weighed. Therefore, these data are all self-reported and prone to bias which may lead to inaccurate results and conclusions [29]. However, it should be noted that the respondents input their weight in the same manner as they were asked during the study period.

## 6. Conclusion

A different approach to managing the obesity epidemic is needed. These data show that, on average, respondents successfully maintained their weight 3 years after completing a 2-year weight loss study. These analyses suggest that, for some individuals, frequent self-weighing appears to be an effective self-guided strategy for long-term weight loss maintenance. Future research and weight maintenance programs may consider the addition of this strategy for long-term weight regain prevention. More studies should be done looking at self-weighing's role and long-term weight gain prevention, particularly the dose-response (i.e., whether weekly weighing is as effective as more frequent weighing). More research should also be done on characteristics of nonresponders in weight management interventions.

## Figures and Tables

**Figure 4:**
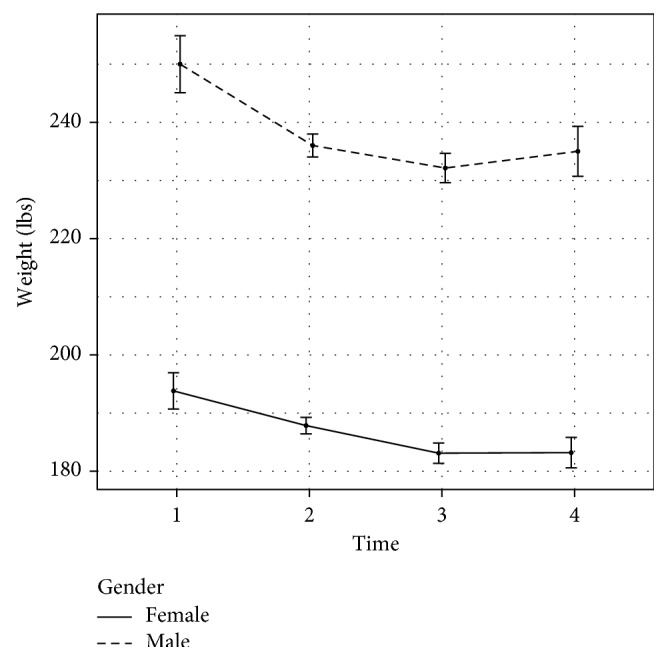
Weight change between baseline and follow-up by gender.

**Table 1:** Follow-up characteristics at the time of the survey.

Follow-up characteristics	
	Total (*n* = 36)
Treatment group, *n* (%)	24 (67%)
Male, *n* (%)	10 (28%)
Age in years, mean (SD)	53.09 (9.78)
Baseline body weight (lbs), mean (SD)	209.42 (37.45)
Baseline BMI, mean (SD)	32.99 (4.69)
Body weight at trial end (lbs), mean (SD)	196.72 (34.97)
Weight change during trial	12.7 (19.4)
Current body weight (lbs), mean (SD)	197.59 (39.31)
Frequency of weighing in days per week during treatment, mean (SD)	6.15 (0.76)
Current frequency of weighing	
Several times per day	0 (0)
1 time/day	8 (22.2%)
Several times/week	12 (33.3%)
Once a week	7 (19.4%)
Less than once a week	7 (19.4%)
Less than once per month	2 (5.6%)
Overall helpfulness of the CTM in reaching long-term weight goal	
Not at all helpful	1 (2.8%)
Slightly helpful	13 (36.1%)
Moderately helpful	12 (33.3%)
Extremely helpful	10 (27.8%)

BMI, body mass index.

**Table 2:** Impact of the CTM on long-term weighing behaviors and weight.

Follow-up characteristics compared to the original sample			
	Declined survey	Accepted survey	*p* value
	Invitation (*n* = 62)	Invitation (*n* = 36)	
Treatment group, *n* (%)	29 (46.8)	24 (66.7)	0.09
Gender (male), *n* (%)	10 (16.1)	10 (27.8)	0.263
Age, mean (SD)	54.08 (8.66)	53.09 (9.78)	0.612
Baseline weight (lbs), mean (SD)	207.51 (40.83)	209.42 (37.45)	0.819
Baseline BMI, mean (SD)	33.94 (5.50)	32.99 (4.69)	0.389
Total change in body weight (lbs) between baseline and year 2, mean (SD)	−4.28 (14.17)	−12.69 (19.40)	0.018^∗^
Frequency of weighing during treatment (times per week), mean (SD)	4.87 (1.86)	6.15 (0.76)	<0.001^∗^
Weekly or more, *n* (%)	53 (91.4)	35 (97.2)	0.489

^∗^Statistical significance.
